# Wearables in Healthcare Organizations: Implications for Occupational Health, Organizational Performance, and Economic Outcomes

**DOI:** 10.3390/healthcare13182289

**Published:** 2025-09-12

**Authors:** Daniele Virgillito, Pierluigi Catalfo, Caterina Ledda

**Affiliations:** 1 Department of Economics and Business, University of Catania, 95100 Catania, Italy; daniele.virgillito@unict.it (D.V.); pierluigi.catalfo@unict.it (P.C.); 2 Department of Clinical and Experimental Medicine, University of Catania, 95123 Catania, Italy; 3 Occupational Medicine Unit, Department of Clinical and Experimental Medicine, University of Catania, 95123 Catania, Italy

**Keywords:** wearable technologies, occupational health, healthcare workers, organizational resilience, staff safety, cost-effectiveness, business continuity, absenteeism

## Abstract

**Background**: Healthcare organizations face major challenges in protecting staff health and ensuring business continuity, particularly in high-risk settings. Wearable technologies are emerging tools to monitor occupational health indicators, improve staff safety, and strengthen organizational resilience. **Objectives**: This scoping review aimed to map the current evidence on wearable technologies in healthcare, focusing on their impact on occupational health, staff safety, and economic outcomes, as well as barriers and facilitators to their adoption. **Methods**: A systematic search was conducted in PubMed, Scopus, Web of Science, Embase, CINAHL, sources from inception to July 2025. Studies were included if they evaluated wearable technologies used by healthcare workers and assessed outcomes related to occupational health, organizational resilience, absenteeism, presenteeism, or cost-effectiveness. The review followed the Arksey and O’Malley framework and PRISMA-ScR guidelines. **Results**: 31 studies met the inclusion criteria. Most were pilot or feasibility studies; only two randomized controlled trials were identified. The wearable technologies evaluated included continuous physiological monitoring devices, real-time location systems, hands-free communication tools, and consumer-grade devices. Evidence suggests potential benefits in improving staff safety, reducing stress and burnout, and enhancing workflow efficiency. However, economic evidence was limited and outcomes varied considerably. Barriers included alert fatigue, privacy concerns, interoperability challenges, and limited staff engagement. Facilitators included leadership support, user-centered design, and adequate infrastructure. **Conclusions**: Wearable technologies show promise for supporting occupational health and organizational resilience in healthcare, but evidence remains fragmented.

## 1. Introduction

Healthcare organizations face significant challenges in protecting staff health and ensuring service continuity in high-risk environments. These include psychological and moral stressors like burnout and ethical dilemmas [1], difficulties with personal protective equipment compliance [2], and violence directly affecting health service delivery [3]. The COVID-19 pandemic has exacerbated these issues, necessitating enhanced infection control measures and occupational health support [4]. Environmental services personnel often lack adequate protection despite high exposure risks [5]. Military and humanitarian organizations face unique challenges in conflict zones and during disease outbreaks [6,7]. Strategies to address these challenges include improving PPE design, implementing comprehensive safety protocols, providing mental health support, and developing preparedness plans for future outbreaks [8].

Occupational health risks in healthcare settings significantly impact organizational performance, patient outcomes, and resilience. Individual and organizational resilience are positively associated with job satisfaction, stress reduction, and improved occupational health [9,10]. Organizational factors, such as work environment and culture, influence worker health, safety, and patient care quality [11,12]. Healthcare workers face unique challenges, including musculoskeletal injuries, which can lead to decreased work effectiveness and potential negative patient outcomes [13]. Employee participation in occupational health and safety practices contributes to organizational resilience, particularly during crises [14]. Resilient healthcare systems exhibit double-loop learning behaviors and adapt to disruptive events [15]. The organizational health framework provides a robust approach to managing employee well-being and organizational performance, emphasizing the link between worker health and productivity [16]. Collectively, health-related absenteeism, presenteeism, and psychosocial risks undermine organizational resilience, service delivery, and economic sustainability [15,16,17].

Healthcare worker illness and absenteeism significantly impact healthcare systems economically. Influenza and respiratory illnesses are common causes, with studies showing high rates of symptoms and missed workdays among healthcare workers [17,18]. Vaccination can reduce absenteeism and associated costs [19,20]. However, presenteeism remains a concern, with many workers continuing to work while ill [17]. The economic burden extends beyond direct wage costs, with productivity losses estimated using wage multipliers ranging from 1.54 to 1.97 [21]. Total economic impact is substantial, with one study estimating $260 billion in lost output annually due to health-related productivity losses across all U.S. workers [22]. Burnout and work stress also contribute to absenteeism in healthcare [23]. Improved management of sickness absence and occupational health services are recommended to address these challenges [24]. However, while their benefits are well documented in industrial sectors, research on wearable technologies in healthcare organizations remains scarce.

While some studies have already explored the use of wearable and visible technologies in organizational contexts, including healthcare, their findings remain limited in scope and fragmented across disciplines [25]. These contributions highlight those wearable solutions are already available, yet their integration faces substantial barriers such as cost, privacy, and organizational readiness.

Our study advances this field by systematically mapping the evidence specific to healthcare organizations, with a focus on how wearables may enhance workforce protection, resilience, and business continuity. Unlike prior contributions, this review integrates occupational health, organizational performance, and economic perspectives, offering a comprehensive view tailored to the healthcare sector.

Wearable technologies offer significant potential for improving staff safety, risk prevention, and data-driven management in various industries, particularly construction and railroads. These technologies include smart helmets with augmented reality [26], physiological sensors [26,27], and exoskeletons [26]. They enable real-time monitoring of workers’ health, environmental conditions, and potential hazards [28,29]. Wearables can enhance personal protective equipment usage [30] and provide data for predictive analytics in occupational safety [27]. Applications include fall detection, exposure evaluation, and musculoskeletal disorder recognition [28]. Integration with connected worker solutions offers contextual information and decision support [27]. These technologies contribute to continuous compliance checking, risk prevention [31], and innovation in risk management systems [32], ultimately improving workplace safety and productivity across various sectors.

Wearable devices are increasingly recognized for their potential to enhance workplace safety, health, and productivity. However, several barriers hinder their adoption, including concerns about employee privacy, compliance, and the cost–benefit ratio [33,34]. Additionally, the lack of employee involvement in device selection and insufficient data demonstrating effectiveness are significant obstacles [27,35]. Facilitators for successful implementation include strong leadership support, effective communication, and a focus on worker-centric approaches [36,37]. Ethical considerations also play a crucial role, as organizations must address potential negative impacts on performance [38]. Overall, understanding these barriers and enablers is essential for integrating wearable technologies into organizational resilience strategies [39].

Although research has examined occupational health risks, organizational resilience, and wearable technologies, the evidence remains fragmented and inconsistent. Most studies focus on single aspects, specific devices, outcomes, or sectors, without providing a comprehensive understanding of how wearables can support workforce protection and business continuity in healthcare.

In particular, little is known about how wearable technologies can be integrated into occupational health services, leveraged to strengthen resilience-oriented strategies, or adapted to sector-specific ethical and organizational challenges.

A scoping review is therefore needed to map the available evidence, clarify key concepts, and identify gaps. This approach will synthesize what is known about (i) the types of wearable technologies used in healthcare, (ii) their impact on staff safety and organizational resilience, and (iii) the barriers and enablers to their adoption. The findings will offer healthcare organizations and decision-makers an integrated perspective to guide future practice and research.

The central research question underpinning this review was how wearable technologies are currently being used to enhance occupational health, staff safety, and organizational resilience in healthcare organizations.

This review is limited to healthcare workers and organizational outcomes, excluding studies that investigated wearable technologies solely for patient-monitoring purposes. Consistent with Arksey & O’Malley and PRISMA-ScR guidance, the objective was framed as the following research question: How are wearable technologies currently being used to enhance occupational health, staff safety, and organizational resilience in healthcare organizations?

## 2. Materials and Methods

This scoping review was conducted following the methodological framework proposed by Arksey and O’Malley [40] and further refined by Levac et al. [41]. Reporting adhered to the Preferred Reporting Items for Systematic Reviews and Meta-Analyses Extension for Scoping Reviews (PRISMA-ScR) guidelines [42].

This methodological framework was considered appropriate as it enables the synthesis of heterogeneous evidence, allows for the identification of knowledge gaps, and provides a structured approach to clarify emerging concepts in a fragmented field such as wearable technologies in healthcare.

In order to ensure methodological transparency and to precisely delineate the scope of the review, the inclusion and exclusion criteria were structured according to the PICOS framework (Population, Intervention, Comparison, Outcomes, Study design) (Table 1). This approach allowed us to systematically define the target population, the types of interventions considered, the relevant comparators, the primary and secondary outcomes of interest, and the eligible study designs, thereby strengthening the consistency and reproducibility of the review process.

### 2.1. Eligibility Criteria

Peer-reviewed articles and gray literature published in English were considered eligible if they:

- addressed the use of wearable technologies in healthcare settings;
- evaluated outcomes related to occupational health, staff safety, organizational resilience, or business continuity;
- examined barriers, facilitators, or economic implications of implementation.

All study designs were eligible, including experimental, observational, qualitative, and mixed-methods studies. Publications focusing solely on patient-monitoring wearables without consideration of staff-related outcomes were excluded. Simulation studies and purely technical validation studies without worker-related outcomes were excluded.

### 2.2. Information Sources and Search Strategy

A comprehensive literature search was conducted across the following databases: PubMed, Scopus, Web of Science, Embase, and CINAHL, from inception to 24 July 2025. Gray literature was identified through Google Scholar and targeted searches of relevant institutional and organizational websites (e.g., World Health Organization, Centers for Disease Control and Prevention, European Agency for Safety and Health at Work).

The search strategy was designed in collaboration with a research librarian and combined free-text keywords and controlled vocabulary (e.g., MeSH terms, Emtree terms) related to three main domains:

- Wearable technologies (e.g., wearable devices, sensors, smart helmets, exoskeletons, biosensors, wearable monitoring systems);
- Healthcare workers (e.g., healthcare staff, clinical personnel, employees, nurses, physicians, allied health professionals, hospital workers);
- Outcomes and organizational aspects (e.g., occupational health, staff safety, workplace safety, organizational resilience, business continuity, absenteeism, productivity).

The final search strings were adapted for the syntax and indexing of each database. The search strategies are reported below:

- PubMed: ((“wearable devices” [MeSH] OR “wearable” [tiab] OR “sensors” [tiab] OR “biosensors” [MeSH] OR “exoskeleton*” [tiab] OR “smart helmet*” [tiab]) AND (“health personnel” [MeSH] OR “healthcare worker*” [tiab] OR “hospital staff” [tiab] OR “nurse*” [tiab] OR “physician*” [tiab] OR “employee*” [tiab]) AND (“occupational health” [MeSH] OR “staff safety” [tiab] OR “workplace safety” [tiab] OR “organizational resilience” [tiab] OR “business continuity” [tiab] OR “absenteeism” [MeSH] OR “productivity” [MeSH])).
- Scopus: (TITLE-ABS-KEY (“wearable device*” OR “wearable” OR “sensor*” OR “exoskeleton*” OR “smart helmet*” OR “biosensor*”) AND TITLE-ABS-KEY (“healthcare worker*” OR “hospital staff” OR “nurse*” OR “physician*” OR “employee*”) AND TITLE-ABS-KEY (“occupational health” OR “staff safety” OR “workplace safety” OR “organizational resilience” OR “business continuity” OR “absenteeism” OR “productivity”)).
- Web of Science: TS = (“wearable device*” OR “wearable” OR “sensor*” OR “exoskeleton*” OR “smart helmet*” OR “biosensor*”) AND TS = (“healthcare worker*” OR “hospital staff” OR “nurse*” OR “physician*” OR “employee*”) AND TS = (“occupational health” OR “staff safety” OR “workplace safety” OR “organizational resilience” OR “business continuity” OR “absenteeism” OR “productivity”).
- Embase: (‘wearable device’/exp OR ‘wearable’:ti,ab OR ‘sensor’/exp OR ‘sensor*’:ti,ab OR ‘exoskeleton’/exp OR ‘exoskeleton*’:ti,ab OR ‘smart helmet*’:ti,ab OR ‘biosensor’/exp OR ‘biosensor*’:ti,ab) AND (‘health care personnel’/exp OR ‘healthcare worker*’:ti,ab OR ‘hospital staff’:ti,ab OR ‘nurse*’:ti,ab OR ‘physician*’:ti,ab OR ‘employee*’:ti,ab) AND (‘occupational health’/exp OR ‘staff safety’:ti,ab OR ‘workplace safety’:ti,ab OR ‘organizational resilience’:ti,ab OR ‘business continuity’:ti,ab OR ‘absenteeism’/exp OR ‘productivity’/exp).
- CINAHL: (“wearable device*” OR “wearable” OR “sensor*” OR “exoskeleton*” OR “smart helmet*” OR “biosensor*”) AND (“healthcare worker*” OR “hospital staff” OR “nurse*” OR “physician*” OR “employee*”) AND (“occupational health” OR “staff safety” OR “workplace safety” OR “organizational resilience” OR “business continuity” OR “absenteeism” OR “productivity”).

For Google Scholar and gray literature, a simplified search was conducted using the combination: “wearable devices” AND “healthcare workers” AND (“occupational health” OR “safety” OR “resilience” OR “business continuity”).

Only studies published in English were considered eligible. All articles retrieved through the search strategy were available in full text and included for screening.

After the initial retrieval of search results, citations from peer-reviewed articles were extracted from the primary databases. To streamline the elimination of duplicates and support the management of the articles selected for inclusion, Rayyan software (https://www.rayyan.ai) was used [43]. Three independent reviewers (P.C., D.V., and C.L.) were involved in the selection process.

The screening was carried out in two phases. In the first phase, two reviewers (P.C. and D.V.) independently assessed the titles, abstracts, and keywords of the articles to evaluate their relevance to the scope of the review. Relevant review articles were also identified for potential citation searching to uncover additional studies not indexed in the selected databases [44]. In the second phase, the same reviewers evaluated the full-text articles according to the inclusion and exclusion criteria. Any disagreements were resolved through consultation with the third reviewer (C.L.), who oversaw the process and ensured consistency across both phases. Inter-rater reliability was not formally calculated. A PRISMA-ScR flow diagram was used to document the selection process. The review protocol is in the process of being registered on the Open Science Framework (OSF), and the registration details will be provided accordingly.

## 3. Results

### 3.1. Literature Search

**Figure 1:**
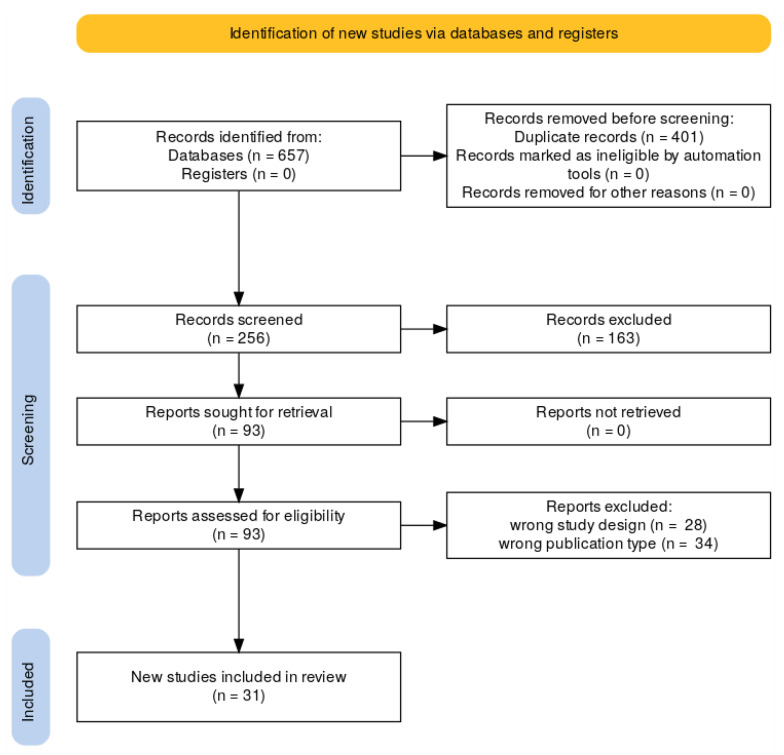
PRISMA flow diagram illustrating the study selection process for the scoping review.

A comprehensive search across several electronic databases initially yielded 657 records. The PRISMA flowchart (Figure 1) details the process of selecting relevant publications at each step of the review. After eliminating 401 duplicate entries, 256 records advanced to the screening phase. During this stage, 163 records were excluded based on their failure to meet the established inclusion criteria. This left 93 articles to be further assessed for eligibility. In the final phase, 31 studies were included in the qualitative/quantitative synthesis.

Among the 69 included studies, pilot or feasibility designs predominated (approximately 40%), followed by observational cohort or cross-sectional studies (about 25%). Qualitative approaches accounted for nearly 15% of the studies, while mixed-methods designs represented around 10%. Randomized controlled trials and case studies/series were less common, each contributing less than 10% of the total. In terms of technology, multiparameter wearable systems such as SensiumVitals, Philips Biosensor/Healthdot, and ViSi Mobile/HealthPatch were the most frequently investigated. Consumer-grade devices including Fitbit and WHOOP bands, as well as specialized solutions such as smart glasses and exoskeletons, were also represented, though less consistently. Overall, the distribution of study types and technologies reflects an exploratory stage of research, with significant heterogeneity across settings and outcomes.

### 3.2. General Characteristics of the Included Studies

A total of 32 studies met the inclusion criteria and were included in this scoping review (Table 2). These studies encompassed a diverse range of designs, healthcare settings, and wearable technologies. The majority were conducted in hospital-based environments, including academic medical centers, surgical wards, intensive care units, emergency departments, pediatric hospitals, dementia care homes, and inpatient units, while a smaller number targeted community-based or multi-center setting.

Economic implications were rarely reported in the included studies. Most publications did not provide direct analyses of cost variables and often listed this domain as “no economic analysis.” A few exceptions emerged: Rosiello et al. [56] documented direct savings of €54–90 per patient through reduced nursing time, and Weenk et al. [52] reported reductions in workload-related costs. Kooij et al. [53] highlighted shortened hospital stays and earlier home recovery associated with biosensor implementation, while Hanna [72] discussed cost as a major barrier to adoption across multiple sensor types. Beyond these cases, economic aspects were generally addressed only indirectly, for example, through references to efficiency gains, workflow changes, or resource optimization. Overall, the scarcity and inconsistency of cost-related outcomes underscore a major gap in the evidence base regarding the economic impact of wearable technologies in healthcare settings.

Regarding study design, most were pilot or feasibility studies [45,46,47,48,49,50,63,67,70,71], frequently adopting longitudinal approaches to evaluate usability, adoption, and implementation outcomes. Observational cohort or cross-sectional studies were also common [58,61,65,68,69,74,75]. Other study designs included randomized controlled trials [52,56], qualitative or mixed-methods evaluations [51,53,55,60,62,64,72,73], case studies [54,66], and case series [66].

The types of wearable technologies evaluated were heterogeneous. Frequently investigated devices included SensiumVitals [58,59,61,75], Philips Biosensor [53], ViSi Mobile and HealthPatch [52], and multiparameter wireless systems such as Wireless Vital Parameter Monitoring (WVPCM) [56] and Healthdot [57]. Consumer-grade devices were also used, including the WHOOP band [45], Fitbit Charge [48,62], and MUSE-S™ [50]. Other technologies included smart glasses [51], proximity beacons [49], real-time location systems [70], wireless sensor systems [67], hands-free communication devices [68], near-field display devices [63], and various multiparameter armbands and biosensors [47,66,72].

Primary outcomes varied but frequently focused on device usability, adoption rate, user satisfaction, and perceived impact on occupational health and safety. High user engagement and satisfaction were reported in studies where devices were user-friendly and minimally disruptive [50,51,59]. Barriers to implementation included digital literacy gaps [48], privacy and ethical concerns [51,54], alert fatigue [61], and infrastructure or interoperability issues [72,73].

Economic outcomes were rarely evaluated, although Rosiello et al. [56] demonstrated cost savings of €54–90 per patient through nursing time reduction, and Weenk et al. [52] reported reductions in workload and costs. Facilitators for successful adoption included user-centered design, leadership support, effective staff training, and adequate IT infrastructure [55,57]. Overall, the evidence suggests that wearable technologies can enhance staff safety, occupational health, and organizational resilience, although variability in study design and heterogeneity in devices and outcomes limit direct comparisons across studies.

#### 3.2.1. Staff Safety

Several studies demonstrated improvements in staff safety through wearable monitoring systems, real-time location devices, and proximity beacons. Reported outcomes included enhanced situational awareness, reduced risk of accidents, and improved compliance with safety protocols. Devices such as location trackers [54,70] and wireless sensor systems [67] were particularly effective in high-risk environments, supporting organizational resilience by ensuring timely interventions.

#### 3.2.2. Stress and Burnout

Wearables capable of monitoring heart rate variability (HRV), sleep quality, and other physiological indicators were frequently employed to detect stress and burnout [45,47,50]. Studies reported reductions in perceived stress and improvements in resilience and quality of life, particularly when devices were user-friendly and integrated smoothly into workflows. However, the predominance of pilot designs limits the generalizability of these findings.

#### 3.2.3. Absenteeism and Presenteeism

Although not commonly primary outcomes, some studies indirectly reported on absenteeism and presenteeism. Improved workload management and early detection of stress symptoms [52,56] suggest potential benefits for reducing sick leave and sustaining staff presence. Nevertheless, robust evidence linking wearables directly to absenteeism reduction remains limited.

#### 3.2.4. Economic and Cost Implications

Economic outcomes were seldom analyzed. A few studies documented measurable benefits: for instance, Rosiello et al. [56] reported direct savings of €54–90 per patient through nursing time reduction, and Weenk et al. [52] described workload-related cost savings. Kooij et al. [53] highlighted reduced hospital stays, while Hanna [72] identified high costs as a barrier to adoption. Overall, cost-effectiveness evidence remains scarce and inconsistent.

### 3.3. Results of Characteristics Referred to PICOS

The characteristics of the included studies were analyzed and summarized according to the PICOS framework (Table 1).

The characteristics of the included studies were analyzed according to the PICOS framework. To enhance clarity and avoid redundancy, the results are summarized in Table 3 using bullet points. This table highlights the main features of the study populations, types of interventions, comparators, outcomes, and study designs.

Population (P). All 32 studies focused on healthcare professionals working in hospital or clinical settings, including nurses, physicians, residents, allied health professionals, and support staff. The majority were conducted in high-pressure environments such as emergency departments, intensive care units, surgical wards, or academic medical centers [45,51,52,56], while others involved pediatric hospitals [55], dementia care homes [54], or general inpatient units [49,69]. The population size varied widely, from small pilot samples of fewer than 20 participants [47,67] to large observational cohorts [58,61].

Intervention (I). The interventions consisted of a wide range of wearable technologies aimed at monitoring physiological parameters, improving communication, or supporting occupational safety. Frequently studied devices included continuous physiological monitoring systems such as SensiumVitals [58,59,61,75], Philips Biosensor [53], ViSi Mobile and HealthPatch [52], and the Wireless Vital Parameter Monitoring (WVPCM) system [56]. Other interventions included consumer-grade devices like the WHOOP band [45], Fitbit [27,48], and MUSE-S™ [50], smart glasses [51], real-time location systems [70], proximity beacons [49], wireless sensor systems [67], hands-free communication devices [68], and near-field display devices [63].

Comparison (C). Most studies compared wearable technologies with standard occupational health and safety practices, while others evaluated different types of wearable devices or implementation strategies. Formal comparative analyses were limited, as many studies were exploratory or descriptive in nature [54,66,72]. Randomized controlled trials [52,56] directly compared wearable monitoring with standard care and demonstrated improvements in workload management and early detection of patient deterioration.

Outcomes (O). Primary outcomes included occupational health and safety metrics, device usability, adoption rates, user satisfaction, stress levels, and organizational resilience indicators. Positive adoption and satisfaction rates were reported when devices were user-friendly and integrated seamlessly into workflows [50,51,58]. Some studies demonstrated improvements in workload management [56,57], staff safety [54,70], and early detection of stress or burnout [45,47]. Secondary outcomes included absenteeism, presenteeism, productivity, and cost implications, though economic evaluations were infrequently performed. Rosiello et al. [56] reported direct cost savings of €54–90 per patient, while Weenk et al. [52] found reductions in workload-related costs.

Study design (S). As anticipated in a scoping review, the included studies were methodologically heterogeneous. Pilot and feasibility studies [46,48,67] predominated, followed by observational cohort or cross-sectional studies [58,59,74], qualitative studies [55,72,73], mixed-methods evaluations [51,62], and randomized controlled trials [52,56]. Sample sizes, outcomes, and study durations varied substantially, reflecting the exploratory nature of the available evidence.

Overall, the findings across all PICOS elements indicate that wearable technologies have the potential to support occupational health, staff safety, and business continuity in healthcare organizations. However, evidence remains fragmented, with significant variability in populations, interventions, and outcome measures, limiting comparability across studies.

## 4. Discussion

This scoping review provides an updated synthesis of the available evidence on wearable technologies used to enhance occupational health, staff safety, and organizational resilience in healthcare settings. The findings from 32 studies demonstrate that wearable devices have the potential to improve early detection of stress and fatigue, support workflow efficiency, and contribute to overall staff safety. However, the evidence base remains fragmented, with most studies focusing on feasibility and usability rather than robust clinical or organizational outcomes. This is consistent with previous reviews in occupational health and safety domains, which have similarly highlighted the exploratory nature of much of the current research on wearables [33,62].

The predominance of pilot and feasibility studies [46,48,50,67] highlights the early stage of evidence development in this area. Randomized controlled trials [52,56] remain scarce but are critical to establishing causal links between wearable device use and improved outcomes. These RCTs reported improvements in workload management and early detection of patient deterioration, aligning with findings from industrial and construction sectors where wearables have been shown to enhance hazard detection and reduce incidents [26,31].

Beyond summarizing results, it is important to critically appraise the evidence base. The predominance of pilot and feasibility studies with small sample sizes and reliance on self-reported outcomes reflects an early stage of evidence development, limiting the generalizability of findings. Few randomized controlled trials and longitudinal follow-ups were available, preventing strong causal inferences or assessment of sustained effects. Similarly, economic evaluations were scarce, with only isolated studies reporting cost savings, leaving a significant gap in understanding cost-effectiveness and long-term return on investment. Ethical and privacy concerns—such as continuous surveillance of staff performance and data governance—were acknowledged in some studies but often insufficiently integrated into their interpretation, raising important questions about acceptability and trust. These gaps mirror those described in previous reviews of occupational safety and digital health, where methodological heterogeneity, limited standardization of outcomes, and ethical challenges were identified as recurrent obstacles to large-scale implementation.

The heterogeneity of devices and outcomes makes it difficult to establish a clear evidence hierarchy. Similar challenges have been reported in previous reviews on digital health technologies, where variability in interventions often impedes meta-analytic synthesis [35,60]. Many studies in our review focused on continuous physiological monitoring devices such as SensiumVitals, Philips Biosensor, and ViSi Mobile, which have been shown to improve early warning detection in patients [58,61]. However, relatively few studies assessed staff-related health outcomes beyond usability and satisfaction, limiting our understanding of their true impact on absenteeism, presenteeism, and organizational resilience.

A key barrier identified was alert fatigue and workflow disruption [58,61], findings consistent with other sectors using real-time alerting systems [29]. Privacy concerns and data governance issues also emerged [51,54], echoing broader ethical concerns around surveillance raised by Maltseva [38] and Biswas et al. [36]. These barriers suggest that the implementation of wearable technologies must be embedded within a broader organizational strategy that emphasizes staff trust and transparent communication, as recommended in other occupational safety frameworks [37].

Wearable technologies show promise in improving occupational health outcomes for healthcare workers. Studies indicate that wearables can help manage stress [76], detect burnout [77], and enhance resilience [78]. These devices enable continuous monitoring of physiological signals, allowing for real-time assessment of work-related stress [47]. Wearables and smartphone apps can facilitate self-care strategies, including mindfulness practices, which have been shown to reduce stress and improve well-being [50,79]. Compared to standard occupational health practices, wearable-based interventions have demonstrated effectiveness in reducing stress, enhancing resilience, and improving quality of life [80]. However, more research is needed to address limitations in data standardization and long-term observations [77]. Overall, wearable technologies offer potential for improving various aspects of healthcare workers’ occupational health, including stress reduction, burnout prevention, and organizational resilience [27].

Wearable technologies in healthcare settings show promise for improving patient outcomes and organizational performance, but evidence of their cost-effectiveness remains limited. Studies indicate that wearables can increase quality-adjusted life years and potentially be cost-saving, depending on factors like device type and health condition [81]. These technologies can enhance employee health, reduce absenteeism, and increase productivity [38,82]. However, implementation challenges include ethical concerns, integration into clinical workflows, and data management [83,84]. While some studies suggest improved productivity and reduced absenteeism in depression management [85], evidence for wearables’ impact on chronic disease outcomes is mixed [86]. More high-quality research is needed to fully understand the long-term benefits and cost-effectiveness of wearable technologies in healthcare settings [81,87].

The synthesis of evidence underscores those wearable technologies alone are unlikely to produce significant improvements unless integrated with systemic organizational change. Studies reporting the most positive results emphasized user-centered design and active staff involvement in device selection and deployment [55,72]. This aligns with evidence from integrated care literature, which identifies leadership support, training, and stakeholder engagement as key enablers of successful workforce innovation [37].

From an occupational health perspective, wearable devices that monitor physiological stress and workload may provide actionable data to inform staffing decisions and targeted interventions [45,47]. Real-time location systems and duress alarms [68,70] have shown promise in enhancing staff safety during critical incidents. These findings mirror the adoption of similar technologies in high-risk industries such as mining and construction, where location-based solutions are widely used to improve situational awareness [28].

Economic evidence, while limited, suggests potential for cost savings through reduced complications and improved workload management [52,56]. However, the lack of full cost–benefit analyses remains a significant gap. Studies outside healthcare have demonstrated positive return-on-investment metrics for wearable technologies when linked to reductions in absenteeism and injury-related costs [31,33]. Similar evaluations are urgently needed in healthcare to support decision-making and large-scale investment.

Recent research highlights the potential of wearable technologies and organizational interventions to improve healthcare workers’ safety, stress levels, and occupational health outcomes. Wearable devices can monitor vital signs, physical activity, and sleep patterns, helping to prevent accidents and manage fatigue [27,77]. These technologies, combined with connected worker solutions, provide real-time insights into workplace safety and productivity [27]. Organizational interventions, such as team development and safety training programs, have been shown to reduce work-related stress [88]. Integration of wearables with health and safety management systems can support worker well-being during technological changes [89]. However, challenges remain, including privacy concerns and the need for improved data standardization [77]. While cognitive-behavioral therapy and relaxation techniques show promise in stress reduction, more research is needed to evaluate the effectiveness of various interventions [90].

Wearable technologies in healthcare settings show promising cost-effectiveness and return on investment profiles. Studies indicate that these devices can increase quality-adjusted life years and be cost-effective or cost-saving, depending on factors such as device type and health condition addressed [81]. Implementing wearable sensors for pressure injury prevention has demonstrated increased turn compliance, decreased hospital-acquired pressure injuries, and reduced organizational costs [91]. A workplace disability management program utilizing wearable technologies showed a 66.6% reduction in sickness absence days and a return on investment of 27.66 [92]. Similarly, a preventive mental health intervention for nurses yielded net savings of 244–651 euros per nurse within six months [93]. Continuous monitoring systems in medical-surgical units have also shown highly positive returns on investment, with potential cost savings ranging from $3,268,000 to $9,089,000 over five years [94].

The evidence reviewed is characterized by methodological heterogeneity and a predominance of small-scale studies. Many relied on self-reported outcomes, which may be subject to response and recall bias. Only two randomized trials were identified [52,56], and both were limited to specific clinical settings, reducing generalizability. Few studies included longitudinal follow-up to assess sustained impact on occupational health or organizational outcomes.

The scarcity of economic evaluations and the limited exploration of ethical issues represent further gaps. Privacy concerns, particularly around continuous monitoring of staff performance, could undermine user acceptance if not adequately addressed [38,54]. There is also a lack of standardized outcome measures, making it difficult to benchmark the effectiveness of different devices or implementation strategies. These gaps mirror those identified in broader digital health implementation research [27,60].

Future research should prioritize high-quality randomized controlled trials and longitudinal cohort studies that evaluate both staff-centered and organizational outcomes, including absenteeism, presenteeism, burnout, and productivity. Mixed-methods approaches will be essential to capture the complex interplay between technology, workflow, and organizational culture.

Further, robust economic evaluations are needed to determine the cost-effectiveness of wearable technologies and to justify their integration into occupational health programs. Addressing ethical considerations, particularly data governance and surveillance concerns, will also be critical to enhancing staff trust and adoption. Finally, international collaboration to develop standardized outcome metrics could facilitate cross-study comparisons and strengthen the evidence base.

Despite the growing potential of wearable technologies in healthcare, several barriers hinder their integration into organizational practice. Concerns regarding data privacy, cost–benefit ratios, and interoperability with existing systems remain key challenges. In addition, issues such as limited digital literacy, alert fatigue, and lack of staff involvement in device selection contribute to resistance. Strategies to foster adoption include strong leadership support, early and transparent communication, dedicated staff training, and worker-centered approaches that prioritize usability and ethical safeguards. Embedding these elements within organizational policies can enhance adherence and long-term sustainability.

This study has several limitations. First, only articles published in English were included, which may have excluded relevant evidence from other languages. Third, the included studies were highly heterogeneous in terms of design, sample size, and outcome measures, limiting comparability and precluding meta-analysis. Nevertheless, this scoping review contributes to the scientific field by offering an integrated overview of wearable technologies in healthcare, emphasizing their links with occupational health, staff safety, and organizational resilience. By identifying both evidence and gaps, the review provides a foundation for future research and policy development.

## 5. Conclusions

This scoping review highlights the emerging but still fragmented evidence on wearable technologies as tools to support occupational health, staff safety, and business continuity in healthcare organizations. While many devices demonstrated potential benefits in reducing stress, preventing burnout, and improving workflow efficiency, the heterogeneity of interventions and outcomes limits definitive conclusions.

From a practical perspective, the findings indicate that wearable technologies can complement traditional occupational health and safety programs by enabling continuous monitoring, supporting early risk detection, and enhancing organizational resilience. However, successful adoption requires addressing challenges such as privacy concerns, staff acceptance, digital literacy, and system interoperability. Ethical considerations, particularly regarding surveillance and worker autonomy, remain critical to ensure that implementation enhances, rather than undermines, trust in healthcare workplaces. The lack of robust economic evaluations further limits understanding of cost-effectiveness, representing a key gap for both researchers and decision-makers.

Future research should prioritize transdisciplinary approaches that combine occupational health, organizational science, and economic evaluation to generate integrated evidence. This will allow for a better understanding of how wearable technologies can deliver sustained improvements in healthcare worker well-being and productivity, while ensuring cost-effectiveness and scalability. Robust randomized controlled trials, longitudinal cohort studies, and standardized outcome measures are essential to advance the field and guide policy and investment decisions.

## Figures and Tables

**Table 1:** PICOS framework summarizing the population, interventions, comparators, outcomes, and study designs included in the scoping review.

Population (P)	Healthcare workers, including nurses, physicians, hospital staff, and allied health professionals, employed in clinical and hospital settings. These individuals operate in high-risk occupational environments characterized by psychosocial stressors, absenteeism, and the need for continuous service delivery.
Intervention (I)	Implementation of wearable technologies (e.g., physiological sensors, biometric patches, smart glasses, real-time location systems, exoskeletons, and continuous monitoring devices). The intervention focuses on enhancing occupational health, staff safety, and organizational resilience through continuous monitoring, predictive analytics, and integration with occupational health protocols.
Comparison (C)	Standard occupational health and safety practices without the use of wearable devices, or alternative types of wearable technologies/implementation strategies.
Outcomes (O)	Primary outcomes: Improvements in staff safety and occupational health, reductions in stress and burnout, enhanced organizational resilience, and business continuity. Secondary outcomes: Reduction in absenteeism and presenteeism, improvements in productivity, acceptance and adoption rates of wearable technologies, user satisfaction, and economic/financial implications of implementation.
Study design (S)	All study designs were eligible, including experimental (e.g., randomized controlled trials, pilot studies), observational (e.g., cohort, cross-sectional, longitudinal), qualitative, and mixed-methods studies.

**Table 2:** General characteristics of the 32 studies included in the scoping review, including setting, study design, population, type of wearable technology, and primary outcomes.

Study	Study Design	Healthcare Setting	Technology Type	Primary Outcomes	Adoption Rate	User Satisfaction	Implementation Challenges	Measured Impact	Cost Implications	Implementation Requirements
Agarwal et al., 2024 [45]	Pilot, Longitudinal	Academic, urban, level 1 trauma center (US)	WHOOP band	Device use, acceptance, burnout, HRV, sleep	50% consistent use	Residents more engaged	Device features, engagement	Device use, mild anxiety, low fulfillment	No economic analysis	Device optimization
Paradiso et al., 2024 [46]	Pilot study	No mention found	Wearable Wellness System (WWS) by Smartex	Heart rate (HR), HRV, stress, posture	No mention found	Designed for comfort	No mention found	Stress, posture	No economic analysis	Comfortable design
Shishavan et al., 2022 [47]	Pilot, Longitudinal	Intensive care unit, no mention found	Multiparameter armband	HRV, pulse transit time (PTT), stress	High	Well accepted	No mention found	Stress detection	No economic analysis	High signal quality
Clingan et al., 2021 [48]	Longitudinal, Pilot	Academic institution (Michigan, US)	Fitbit Charge 3, TempTraq	Feasibility, HR, temperature, COVID-19 detection	No mention found	No mention found	Internet access, digital literacy	Feasible, mood/symptom tracking	No economic analysis	Digital literacy
Keller et al., 2021 [49]	Pilot study	Inpatient unit, no mention found	Proximity beacons	No mention found	No mention found	No mention found	Feasibility, physical distancing	No mention found	No economic analysis	Feasibility
Ghosh et al., 2023 [50]	Pilot study	No mention found	MUSE-S™	Stress, resilience, quality of life (QoL), cognition	High	91.9% felt relaxed, 73% would continue	No mention found	Improved stress, resilience	No economic analysis	Ease of use
Park and Lee, 2025 [51]	Mixed-methods, pretest-posttest	Emergency department	Smart glasses	Communication clarity, workload	No mention found	Improved communication	User interface, privacy, workload	Improved clarity, workload	No economic analysis	Training, privacy
Weenk et al., 2019 [52]	Randomized controlled trial (RCT)	University hospital (Netherlands)	ViSi Mobile, HealthPatch	Psychological distress, usability	No mention found	Generally positive	Device size, training	Positive, reduced workload	Reduced workload, costs	Training, monitoring
Kooij et al., 2022 [53]	Qualitative	Teaching hospitals (Netherlands)	Philips Biosensor, SensiumPatch	No mention found	No mention found	Mixed	Sensor quality, Wi-Fi, workload	Mixed, reduced stays	Reduced stays, home recovery	Interoperability
Scott, 2008 [54]	Case study, qualitative	Dementia care homes (UK)	Location-tracking devices	No mention found	No mention found	No mention found	Privacy, alarms, training	Enhanced safety	No economic analysis	Stakeholder involvement
O’Dwyer et al., 2024 [55]	Qualitative	Pediatric hospital (Canada)	No mention found	No mention found	No mention found	High satisfaction	Risk assessment, staffing	Positive, feasible	No economic analysis	Human-centered design
Rosiello et al., 2021 [56]	Pilot, RCT	Multiple hospitals, no mention found	Wireless Vital Parameter Monitoring (WVPCM) system	Clinical-economic impact	No mention found	No mention found	Nursing time reduction	Reduced complications, readmissions	€54-90 saved per patient	Nursing time reduction
Schonck et al., 2025 [57]	Observational, Longitudinal	Surgical ward, no mention found	Healthdot (Philips)	Workload (Individual Workload Scale), implementation outcomes	No mention found	Assessed via System Usability Scale, Evidence-Based Practice Attitude Scale	Workload, complexity	No mention found	No economic analysis	Efficiency, workload
Joshi et al., 2021 [58]	Cross-sectional	Academic hospital (UK)	SensiumVitals	No mention found	No mention found	High engagement	Alerts, workload	No mention found	No economic analysis	Alerts, comfort
Iqbal et al., 2021 [59]	Pre-post, Observational	National Health Service (NHS) hospital (UK)	SensiumVitals	Clinical outcomes	No mention found	No mention found	No mention Found	No mention found	No economic analysis	Integration, training
Brewster et al., 2014 [60]	Mixed-method review	No mention found	Telehealth	No mention found	No mention found	No mention found	Service change, technical	No mention found	No economic analysis	Service change
Iqbal et al., 2022 [61]	Observational cohort	Surgical unit (UK)	SensiumVitals	Clinical outcomes	No mention found	Favorable	Alert fatigue, workflow	No mention found	No economic analysis	Workflow, policies
Patel et al., 2022 [62]	Mixed methods	General Medicine (Canada)	Fitbit Charge 2/3	No mention found	No mention found	Patients positive, nurses mixed	Device design workflow	No mention found	No economic analysis	Device design
Santomauro et al., 2019 [63]	Pilot study	Rural/regional hospitals	Near-field display devices	No mention found	No mention found	Positive	Functionality preferences	No mention found	No economic analysis	Usability testing
Shah et al., 2021 [64]	Case study, qualitative	Academic hospital (Canada)	Medly (mobile)	No mention found	No mention found	No mention found	Siloed systems workflow	No mention found	No economic analysis	Leadership, connectivity
Buss et al., 2023 [65]	Qualitative evaluation	Academic hospital (UK)	VitalPatch, WristOx2	No mention found	No mention found	Supportive over time	Device numbers contact	Supportive, workload	No economic analysis	Device numbers
Meltzer et al., 2020 [66]	Case series	Academic hospital (US)	IMUs	Ergonomic risk	No mention found	Encouraged	Sample size	Risk factors identified	No economic analysis	Mindfulness
Claudio et al., [67]	Pilot study	Emergency departments	Wireless sensor system	No mention found	No mention found	High	No mention found	Positive	No economic analysis	User feedback
Carreon and Dutra, 2020 [68]	Observational no mention found)	No mention found	Hands-free communication device	No mention found	No mention found	No mention found	Communication failures	Improved workflow	No economic analysis	Infrastructure
Yesmin et al., 2022 [69]	Longitudinal	Hospital ward (Canada)	No mention found	Patient safety outcomes	No mention found	Positive	Training, device issues	Improved care time	No economic analysis	Training, device
Joshi et al., 2022 [58]	Interview study	Academic hospital (UK)	SensiumVitals	No mention found	No mention found	High engagement	Equipment, staff	No mention found	No economic analysis	Comfort, future
Jones et al., 2023 [70]	Pilot study (no mention found)	Academic medical center (US)	Real-time location system (RTLS)	No mention found	No mention found	No mention found	No mention found	Improved safety	No economic analysis	Real-time location system rollout
Jovanov et al., 2011 [71]	Pilot study	Academic nursing (US)	UAHealth (iPhone, Ant+)	Stress index, HRV	No mention found	Facilitated by user interface	No mention found	Stress index, heart rate variability	No economic analysis	User interface, support
Hanna, 2020 [72]	Qualitative study	Multiple (US)	Multiple sensor types	No mention found	No mention found	No mention found	Cost, communication	No mention found	High cost barrier	Collaboration
Mclean et al., 2024 [73]	Qualitative implementation	Tertiary hospitals (UK)	No mention found	No mention found	No mention found	Mixed	Information technology, workload	No mention found	No economic analysis	Information technology, strategy
Lokmic-Tomkins et al., 2025 [74]	Cross-sectional	Clinical settings (Australia)	No mention found	No mention found	No mention found	No mention found	Infrastructure, support	No mention found	No economic analysis	Infrastructure, support

**Table 3:** Summary of included studies according to the PICOS framework.

Element	Summary (Bullet Points)
Population (P)	• Healthcare professionals (nurses, physicians, residents, allied health staff, support staff). • Predominantly hospital and clinical settings (e.g., ICUs, surgical wards, EDs, academic hospitals). • Smaller number in community care, dementia homes, pediatric hospitals.
Intervention (I)	• Continuous physiological monitoring systems (e.g., SensiumVitals, Philips Biosensor/Healthdot, ViSi Mobile/HealthPatch, WVPCM). • Consumer-grade wearables (Fitbit, WHOOP, MUSE-S™) • Specialized technologies (smart glasses, proximity beacons, real-time location systems, exoskeletons, wireless sensors).
Comparison (C)	• Standard occupational health and safety practices. • Alternative wearable devices or implementation strategies. • Limited number of formal comparative analyses.
Outcomes (O)	• Primary: Staff safety, occupational health, stress/burnout reduction, organizational resilience, workflow efficiency. • Secondary: Absenteeism/presenteeism, productivity, adoption and satisfaction, usability, cost implications.
Study Design (S)	• Pilot/feasibility studies (~40%). • Observational cohort or cross-sectional (~25%). • Qualitative (~15%). • Mixed-methods (~10%). • RCTs and case studies/series (<10%).

## Data Availability

Not applicable.
